# Nocebo Effect on Pain‐Related Autonomic Responses in a State of Experimentally‐Induced Sensitization

**DOI:** 10.1002/ejp.70029

**Published:** 2025-04-18

**Authors:** Florin Allmendinger, Jan Rosner, Thomas Egger, Paulina Simonne Scheuren, Michèle Hubli

**Affiliations:** ^1^ Spinal Cord Injury Center Balgrist University Hospital, University of Zurich Zurich Switzerland; ^2^ Danish Pain Research Center, Department of Clinical Medicine Aarhus University Aarhus Denmark; ^3^ International Collaboration on Repair Discoveries University of British Columbia Vancouver British Columbia Canada; ^4^ Department of Anesthesiology, Pharmacology & Therapeutics, Faculty of Medicine University of British Columbia Vancouver British Columbia Canada

## Abstract

**Background:**

Enhanced pain‐related autonomic responses were reported after experimentally‐induced secondary mechanical hyperalgesia (SMH) in healthy individuals as well as in a variety of chronic pain cohorts. Stimulus‐induced autonomic responses can also be modulated by positive and negative expectations towards the applied stimulus. This study aimed to investigate the influence of negative expectations on pain‐related autonomic responses after experimentally‐induced SMH.

**Methods:**

Forty healthy participants (20 females) were recruited and assigned to a NOCEBO or a NAïVE group. Phasic skin conductance responses (SCR) and tonic background skin conductance level (SCL) were recorded in response to 10 pinprick stimuli applied to both volar forearms. On one arm, all stimuli were applied (EXP‐arm) before (PRE) and after (POST) an experimental heat pain model to induce SMH. The other arm served as the control (CTRL‐arm). The NOCEBO group was instructed that the stimuli will be ‘more intense and painful’ in the POST‐assessment. The NAïVE group did not receive any instructions. Pain ratings were matched to a numeric rating scale 4 across all assessments to control for subjective pain perception.

**Results:**

Only the combination of induced SMH and negative expectation (i.e., EXP‐arm in the NOCEBO group) increased the pinprick‐evoked phasic SCRs (*p* < 0.001) from PRE to POST. Tonic background SCL increased from PRE to POST (*p* < 0.01) independent of stimulation area (i.e., EXP‐arm or CTRL‐arm) or group (i.e., NOCEBO or NAïVE).

**Conclusions:**

These results demonstrate facilitatory effects of top‐down modulatory processes (i.e., negative expectations) on pain‐related autonomic responses after experimentally‐induced SMH.

**Significance:**

This study showed a facilitatory effect of negative expectation on enhanced pain‐related autonomic responses in a state of experimentally‐induced sensitisation in healthy participants. Hence, pain‐related autonomic responses are shaped by both bottom‐up (nociceptive input) and top‐down (expectation) modulatory processes. This leads to the clinical implication that increased pain‐related autonomic responses reported in individuals with chronic pain might not solely reflect pain hypersensitivities through nociceptive sensitisation, but also exaggerated negative expectation.

## 1 Introduction

While the perception of pain is often measured on a numeric rating scale (NRS) or a visual analog scale (VAS) (Karcioglu et al. 2018), autonomic responses can also be investigated, for example, by pain‐induced changes in skin conductance (SC), heart rate variability (HRV), blood pressure or pupil dilation (Kyle and McNeil 2014).

By definition, the subjective experience of pain is influenced by biological, psychological and social factors (Raja et al. 2020) and both perceptual (Atlas et al. 2010; Colloca et al. 2010) and autonomic responses to noxious stimuli (i.e., skin conductance responses (SCR)) (Barnes et al. 2021; Koban and Wager 2016; Reicherts et al. 2016) can be modulated by preceding pain experiences and expectations, as well as by observing other people experiencing pain (Hunter et al. 2014). Pain‐related autonomic responses are also increased after experimentally‐induced secondary mechanical hyperalgesia (SMH) (Salameh et al. 2022; Scheuren et al. 2020; van den Broeke et al. 2019) as well as in a variety chronic pain cohorts. These include widespread neuropathic pain after spinal cord injury (Lütolf et al. 2022), migraine (Ozkul and Ay 2007), Parkinson's patients with central pain (Schestatsky et al. 2007) and complex regional pain syndrome (Scheuren et al. 2022), in which central sensitisation is discussed as an underlying mechanism of chronic pain. As experimental models inducing SMH reflect key features of central sensitisation (Quesada et al. 2021), enhanced pain‐related autonomic responses were proposed as potential objective surrogate marker for nociceptive sensitisation (Scheuren et al. 2020; van den Broeke et al. 2019). Additionally, we recently showed that enhanced pinprick‐induced SCRs after experimentally‐induced hyperalgesia are not simply related to higher stimulus‐associated arousal (i.e., increased pinprick sensitivity in the area of SMH) but rather to a general priming of the ANS (Scheuren et al. 2023). Heightened autonomic arousal, measured as increased SCR, was also shown to be correlated with anticipatory anxiety and seems to be a key mechanism for the persistence of nocebo‐induced hyperalgesia (Colagiuri and Quinn 2018). Furthermore, induced negative expectation increased the area of SMH (Jaltare et al. 2024) and the sensitivity of pinprick stimuli applied therein (van den Broeke et al. 2014). To what extent induced negative expectations modulate pain‐related autonomic responses after experimentally‐induced SMH remains, however, unknown. Understanding this contribution would be of importance when using pain‐related autonomic responses as surrogate marker for nociceptive sensitisation in individuals with chronic pain.

Therefore, the aim of this study was to investigate the influence of verbally induced negative expectation on pain‐related autonomic responses after experimentally‐induced SMH in healthy participants. For this purpose, pinprick‐induced phasic SCRs and tonic background skin conductance level (SCL) were recorded during stimulus application within the area of SMH. The intensity of the pinprick stimuli was matched to an NRS 4 to control for stimulus‐associated arousal. We hypothesised that negative expectations will lead to a further enhancement of pinprick‐induced autonomic responses (i.e., SCR and SCL) in the area of SMH.

## 2 Materials and Methods

### 2.1 Participants

Healthy participants, both females and males, between 18 and 40 years of age were recruited for the study and pseudo‐randomly assigned to either a NOCEBO (*N* = 20, 10 females) or a NAïVE group (*N* = 20, 10 females) (see Section 2.6 Nocebo Paradigm). Exclusion criteria were pregnancy, neurological diseases (e.g., polyneuropathy or epilepsy), acute and chronic pain, the intake of analgesic medication within 24 h before the visit, and regular intake of analgesic medication (e.g., antidepressants, opioids, benzodiazepines or anticonvulsants). In addition, all participants were asked to refrain from physical exercise and the intake of alcohol, nicotine and caffeine twelve hours prior to the study visit, as these can affect autonomic responses (Boucsein et al. 2012).

Written informed consent was obtained from all participants prior to any assessment. The study was approved by the local ethics committee ‘Kantonale Ethikkommission Zürich’ (EK‐04/2006, PB_2016‐02051) and conducted in accordance with the Declaration of Helsinki.

### 2.2 Study Design

**FIGURE 1:**
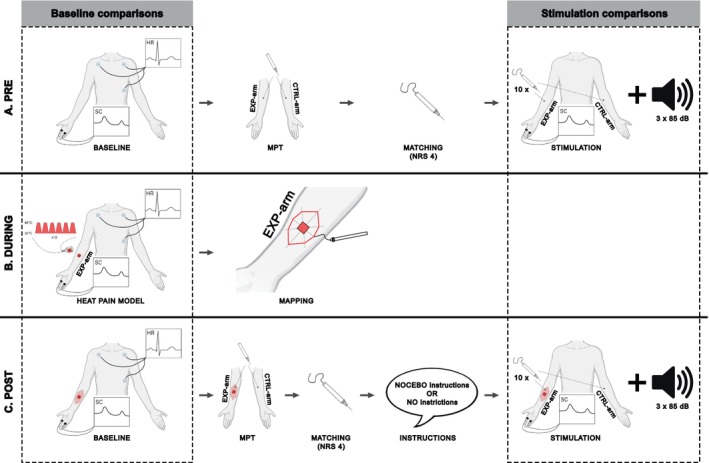
Study design. (a) The PRE‐assessment consisted of a 2‐min measurement of skin conductance (SC) and heart rate (HR), followed by assessment of the mechanical pain threshold (MPT). Then, the pinprick intensity leading to a rating of 4/10 on a numeric rating scale (NRS) was assessed for each participant. Afterwards, SC and pain ratings were recorded in response to 10 pinprick stimuli. This procedure was applied to both forearms. Lastly, SC was recorded in response to 3 acoustic stimuli. (b) SC and HR were recorded during the application of the heat pain model. The area of secondary mechanical hyperalgesia (SMH) was mapped after the heat pain model. (c) In the POST‐assessment, the measurements of the PRE‐assessment were repeated. However, the NOCEBO group received the verbal nocebo instructions before application of the 10 pinprick stimuli, while the Naïve group did not receive further instructions (partially created with BioRender.com).

The study consisted of one three‐hour visit. First, participants were asked to complete a short questionnaire assessing their medical history to ensure the inclusion of participants without any major health issues. Additionally, the Pain Catastrophizing Scale (PCS) (Sullivan et al. 1995) to assess catastrophic thinking in the context of pain and the Hospital Anxiety and Depression Scale (HADS) (Stern 2014) to assess levels of anxiety and depression of the participants PCS and HADS scores were used to investigate homogeneity between the two groups (i.e., NOCEBO and NAïVE). Further, the visit consisted of one assessment before (PRE) and one assessment 20 min after (POST) the experimental induction of SMH using a repetitive heat pain model (Jürgens et al. 2014) on the EXP‐arm. The assignment of the EXP‐arm and the CTRL‐arm (i.e., left or right arm) was randomised a priori for each participant. Each assessment involved a two‐minute baseline measurement followed by an assessment of the mechanical pain threshold (MPT) and a sequence of 10 pinprick stimuli applied to both volar forearms (i.e., EXP‐arm and CTRL‐arm) as well as 3 acoustic stimuli. The MPT and sequence of pinprick stimuli were applied to the expected area of SMH, that is, adjacent to the area of the conditioning stimulus, (PRE) and the actual area of SMH (POST) on the EXP‐arm and the mirrored area on the CTRL‐arm. The acoustic stimuli were randomly applied either after pinprick stimulation of the EXP‐arm or the CTRL‐arm in the PRE and POST assessments. The MPT was assessed to capture the development of SMH after the heat pain model. Skin conductance (SC) and heart rate (HR) were recorded during both baseline measurements and the first block of the heat pain model to investigate general autonomic arousal and its change due to the applied heat pain model. In addition, SC was recorded during all pinprick and acoustic stimulation sequences, allowing the exploration of nocebo and sensitisation effects on stimulus‐related phasic SCR and tonic SCL. For a detailed study design see Figure 1.

### 2.3 Experimentally‐Induced Secondary Mechanical Hyperalgesia

After the PRE assessment, a repetitive phasic heat pain model (Jürgens et al. 2014) was used to induce SMH on the EXP‐arm. Noxious heat stimuli were applied to the volar forearm of the EXP‐arm with a 30 × 30 mm Advanced Thermal Stimulation (ATS) thermode (PATHWAY Pain and Sensory Evaluation System, Medoc Ltd., Ramat Yishai, Israel). The paradigm consisted of 10 blocks of 6 noxious heat stimuli (baseline: 32°C; target: 48°C; duration 6 s; ramp 10°C/s; no interstimulus interval (ISI): 0 s; interblock interval: 30 s). After each block, participants were asked to rate the intensity of the block on a numeric rating scale (NRS) from 0 (‘no pain’) to 10 (‘worst pain imaginable’).

Twenty minutes after the heat pain model, a calibrated 256 mN von Frey filament with a rounded tip (0.5 mm in diameter) (OptiHair2, MRC Systems, Germany) was used to map the area of SMH (Jürgens et al. 2014). Starting from eight different angles approximately 10–15 cm outside the center (i.e., 30 × 30mm surface of the ATS thermode), von Frey stimuli were applied in 5 mm steps. Participants were instructed to indicate the point they felt a clear change in sensation from touch to pain. All eight points were marked on the skin, transferred to a transparent sheet and scanned for further analysis. The ‘Measure’ tool within Adobe Acrobat Reader DC was used to quantify the area of SMH in cm^2^.

### 2.4 Mechanical Pain Threshold

The MPT was assessed according to the Quantitative Sensory Testing (QST) battery developed by the German Network on Neuropathic Pain (Rolke et al. 2006) to detect mechanical hyperalgesia, using weighted pinprick stimulators (8–512 mN; MRC Systems, Heidelberg, Germany). The MPT was assessed in a predefined area on the volar forearm, adjacent to the area where the ATS thermode was placed for the repetitive heat pain model. This area corresponded to the area of SMH on the EXP arm in the POST assessment and the mirrored area on the CTRL arm. The dorsal side of the non‐dominant hand was used for familiarisation prior to testing.

### 2.5 Stimulation

#### 2.5.1 Pinprick Stimulation

The intensity of all pinprick stimuli was matched to an NRS 4 out of 10 for all participants across both assessments (PRE/POST) and both testing areas (CTRL‐arm/EXP‐arm) prior to the respective stimulation sequence (Scheuren et al. 2023). For this purpose, different pinprick forces (8–512 mN) were applied, starting at 8 mN, and repeatedly increased until participants rated the stimulus as NRS 4.

Prior to each stimulation sequence, participants were asked to indicate how painful they expected the following stimulation sequence to be (NRS 0–10). A stimulation sequence consisted of 10 pinprick stimuli applied in the predefined area on the volar forearm, which corresponded to the area of SMH of the EXP‐arm in the POST assessment and the mirrored area of the CTRL‐arm. Each pinprick stimulus generated a trigger signal, allowing for time‐locked analysis of stimulus‐related responses. The interstimulus interval was 13–17 s. The pinprick stimulator was repositioned slightly after each stimulus, ensuring a single point was not stimulated twice in a row. An auditory cue signalled the participants to give a rating of the perceived pain on an NRS (0–10) nine seconds after each stimulus.

#### 2.5.2 Acoustic Stimulation

Three acoustic stimuli were given after pinprick stimulation of the EXP‐arm or the CTRL‐arm in the PRE and POST assessment. An acoustic stimulus consisted of a single auditory outburst (1000 Hz, 200 ms, 85 dB) (Boettger et al. 2010) delivered by two loudspeakers (TEAC, PowerMax 60/2, Stereo Speaker System). Each acoustic stimulus generated a trigger signal, allowing for time‐locked analysis of stimulus‐related responses. The interstimulus interval was 13–17 s.

### 2.6 Nocebo Paradigm

The NOCEBO group was verbally given the instruction (in German): ‘The following stimulations will be more intense and painful than in the first assessment’ (‘Nachfolgende Stimulationen werden intensiver und schmerzhafter sein, als jene zu Beginn der Studiensitzung’) prior to each stimulation sequence in the POST assessment. The NAïVE group did not receive any further instructions.

### 2.7 Recording of Autonomic Responses

SC and HR were recorded with the data acquisition hardware PowerLab (ADInstruments, Dunedin, New Zealand) and the corresponding software LabChart Pro (v.8.1.13, ADInstruments, Dunedin, New Zealand). SC was recorded at a sampling frequency of 1000 Hz with two bipolar finger electrodes (MLT118F GSR Finger Electrodes, ADInstruments, Dunedin, New Zealand) attached to the palmar surface of the index and ring finger of the EXP‐arm and amplified by a galvanic skin response amplifier (FE116 GSRamp, ADInstruments, Dunedin, New Zealand). PowerLab records SC change in a recording window from −40 μS to +40 μS. An initial baseline correction of SC was performed for each participant to ensure measuring the absolute SC change within the given recording window. The conductivity value of the baseline correction was post hoc added to recorded values of SC for data analysis. If the SC still exceeded this recording window during a recording session, this trial was retrospectively excluded from further analysis.

An electrocardiogram (ECG) was recorded with a three‐lead recording set‐up using disposable Ag/AgCl electrodes (Kendall H124SG Elektrode, Cardinal Health, Germany). Electrodes were attached below the left (negative) and right (ground) clavicles and below the left rib cage (positive). Electrode skin sites were prepared with alcohol (Softasept N, B. Braun Medical AG, Germany) to ensure low impedance. The signal was band‐pass filtered between 0.3 and 1000 Hz and sampled at 1000 Hz with the Dual Bio Amp (FE232, ADInstruments, Dunedin, New Zealand). ECG recordings were visually inspected for abnormalities and excluded from further analysis if needed.

### 2.8 Data Processing

An open‐source Matlab software (Ledalab V3.4.9) was used for the analysis of the SC data. A continuous decomposition analysis (Benedek and Kaernbach 2010) of the SC data was performed to separate tonic background SCL and phasic SCR. SCL and SCR were analysed in a 1‐to‐7s post‐trigger window after each pinprick/acoustic stimulus. SCL was defined as mean tonic activity within the 1‐to‐7s time window and the SCR was defined as the time integral of the phasic driver reflecting the cumulative phasic activity within this time window. The mean of all 10 pinprick‐induced and three acoustic‐induced SCRs and SCLs was used for further analyses. In addition, the mean SCL during (1) the 2‐min baseline in the PRE assessment, (2) the 1‐min window of the first block of the heat pain model (DURING) and (3) the 2‐min baseline in the POST assessment was extracted.

Heart rate variability (HRV) was analysed using R statistical software (R version 4.2.2 for Windows) applying the R package ‘RHRV’. HR, root mean square of successive differences (RMSSD) and the ratio of low‐frequency power to high‐frequency power (LF/HF ratio) was calculated during (1) the 2‐min baseline in the PRE‐assessment, (2) the 1‐min window of the first block of the heat pain model (DURING) and (3) the 2‐min baseline in the POST assessment.

### 2.9 Statistics

The differences in age and questionnaire scores (i.e., PCS, HADS) between the NOCEBO and NAÏVE groups were assessed with Wilcoxon signed‐rank tests to detect potential underlying group differences.

Two linear mixed models (‘lmer’ function of the R package ‘lme4’) were used to assess the effect of ‘time’ (PRE and POST) and ‘area’ (CTRL‐arm and EXP‐arm) on (1) MPT and (2) stimulation intensity needed to reach NRS 4. The interaction ‘time × area’ was included in each model and ‘participant’ was included as a random effect. Post hoc multiple comparisons (R package ‘emmeans’) were performed on the interaction ‘time × area’ to detect area‐specific differences before and after experimentally‐induced SMH.

To assess the effect of ‘time’ (PRE and POST) and ‘group’ (NOCEBO and NAïVE) on (1) expected pain intensity, (2) pain intensity, (3) SCR and (4) SCL, we used separate linear mixed models (‘lmer’) for the CTRL‐arm and EXP‐arm. The interaction ‘time × group’ was included in all models and ‘participant’ was included as a random effect. Non‐significant interactions ‘time × group’ were removed from the models. Post hoc multiple comparisons (‘emmeans’) were performed on significant interactions ‘time × group’.

Two linear mixed models (‘lmer’) assessed the effect of ‘time’ (PRE and POST) and ‘group’ (NOCEBO and NAïVE) on (1) SCR and (2) SCL of acoustic stimulation. The interaction ‘time × group’ was included in all models and ‘participant’ was included as a random effect. Non‐significant interactions ‘time × group’ were removed from the models. Post hoc multiple comparisons (‘emmeans’) were performed on significant interactions ‘time × group’.

Four linear mixed models (‘lmer’) were used to analyse the effect of ‘time’ (PRE, DURING and POST) on (1) SCL, (2) HR, (3) RMSSD and (4) LF/HF ratio during the two baseline measurements and the heat pain model. ‘Participant’ was included as a random effect in both models. Post hoc multiple comparisons (‘emmeans’) were performed on the factor ‘time’.

All statistical analyses were performed in R statistical software (R version 4.2.2 for Windows). Fulfilment of model criteria were checked with diagnostic plots (i.e., quantile‐quantile plots and histograms). Significance level was set to *p* < 0.05. Bonferroni corrections were performed to adjust for multiple comparison.

## 3 Results

In the following results section, differences of the various readouts between timepoints (i.e., PRE and POST) and groups (i.e., NAïVE and NOCEBO) within the stimulated areas (i.e., CTRL‐arm and EXP‐arm) are reported. Full model statistics, including *F*‐ and *p*‐values for main‐ and interaction‐effects, as well as effect sizes Hedges' *g* of post hoc comparisons can be found in the Supporting Information Tables S1–S5. Further, a table with the numbers of exclusions of SC values due to exceeding the recording window of ±40 μS can be found in the Supporting Information Table S6.

### 3.1 Demographics

Forty healthy participants (20 females) were recruited. The NOCEBO (25.0 ± 1.7 years) and the NAïVE group (25.3 ± 1.6 years) did not differ in age (*N* = 40; *W* = 225; *p* = 0.50). Furthermore, neither the PCS scores (Nocebo: 15.5 ± 8.6; NAïVE: 11.8 ± 7.2; *N* = 40; *W* = 146.5; *p* = 0.15), nor the HADS anxiety scores (NOCEBO: 4.4 ± 3.4; NAïVE: 3.9 ± 2.1; *N* = 40; *W* = 192; *p* = 0.84) or HADS depression scores (NOCEBO: 1.6 ± 1.7; NAïVE: 1.0 ± 1.3; *N* = 40; *W* = 162.5; *p* = 0.29) differed between the two groups.

### 3.2 Experimentally‐Induced Secondary Mechanical Hyperalgesia

The applied heat pain model was perceived as painful by all participants (7.5 ± 1.3 NRS) and led to the development of a pronounced area of SMH (66.5 ± 23.9 cm^2^) on the EXP‐arm. The heat pain model had a significant effect on the MPT (‘time × area’; *F* = 4.24; *p* = 0.04) and the stimulation intensity needed to reach NRS4 (‘time × area’; *F* = 61.45; *p* < 0.001).

**FIGURE 2:**
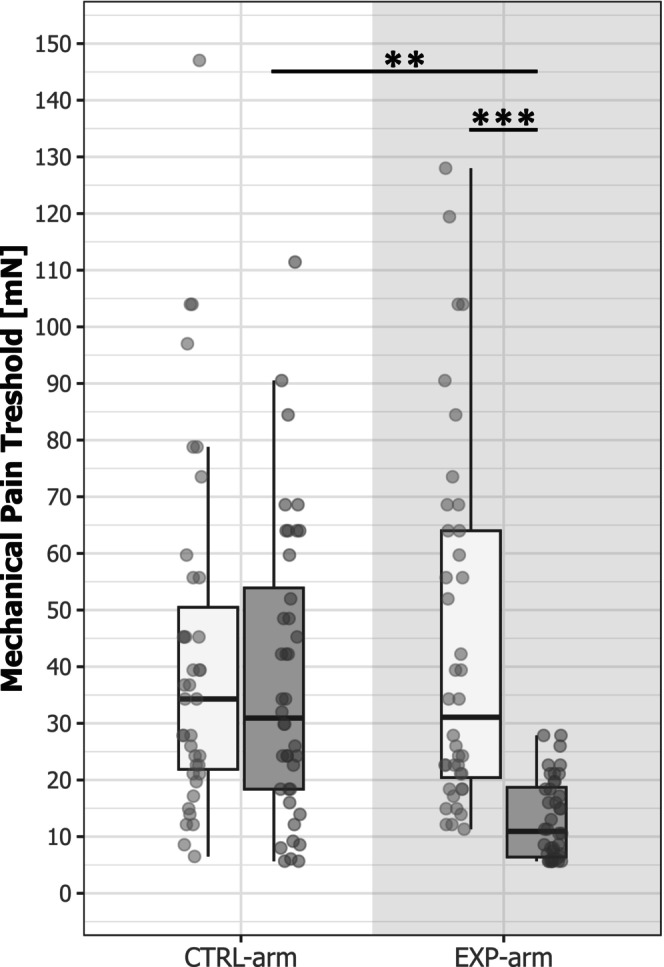
Mechanical pain threshold (MPT). The MPT decreased from PRE (light) to POST (dark) on the EXP‐arm but not on the CTRL‐arm. **p < 0.01; ***p < 0.001.

Post hoc comparisons revealed that the MPT of the EXP‐arm decreased from PRE (44.2 ± 32.0 mN) to POST (13.0 ± 7.1 mN; *N* = 40; *t* = −4.72; *p* = 0.001; Hedges' *g* = −1.10), while there was no change in the MPT of the CTRL‐arm (PRE: 49.6 ± 55.8 mN; POST: 37.7 ± 25.7 mN; *N* = 40; *t* = −1.81; *p* = 0.29; Hedges' *g* = −0.26) (Figure 2). Similarly, the pinprick intensity needed to elicit an NRS 4 decreased on the EXP‐arm from PRE (451.2 ± 126.3 mN) to POST (193.0 ± 169.8 mN; *N* = 40; *t* = −11.98; *p* < 0.001; Hedges' *g* = −1.67) but not on the CTRL‐arm (PRE: 470.4 ± 102.0 mN; POST: 451.2 ± 115.9 mN; *N* = 40; *t* = −0.89; *p* = 1.0; Hedges' *g* = −0.17). Both the change in MPT and applied pinprick intensity on the EXP‐arm provide psychophysical evidence for the development of SMH after the heat pain model, but not on the CTRL‐arm.

### 3.3 Pinprick Stimulation

#### 3.3.1 Increased Expected Pain Intensity in the NOCEBO Group

The expected pain intensity varied depending on the group (NOCEBO/NAïVE), both in the EXP‐arm (‘time × group’; *F* = 12.30; *p* = 0.001) and the CTRL‐arm (‘time × group’; *F* = 5.85; *p* = 0.02).

**FIGURE 3:**
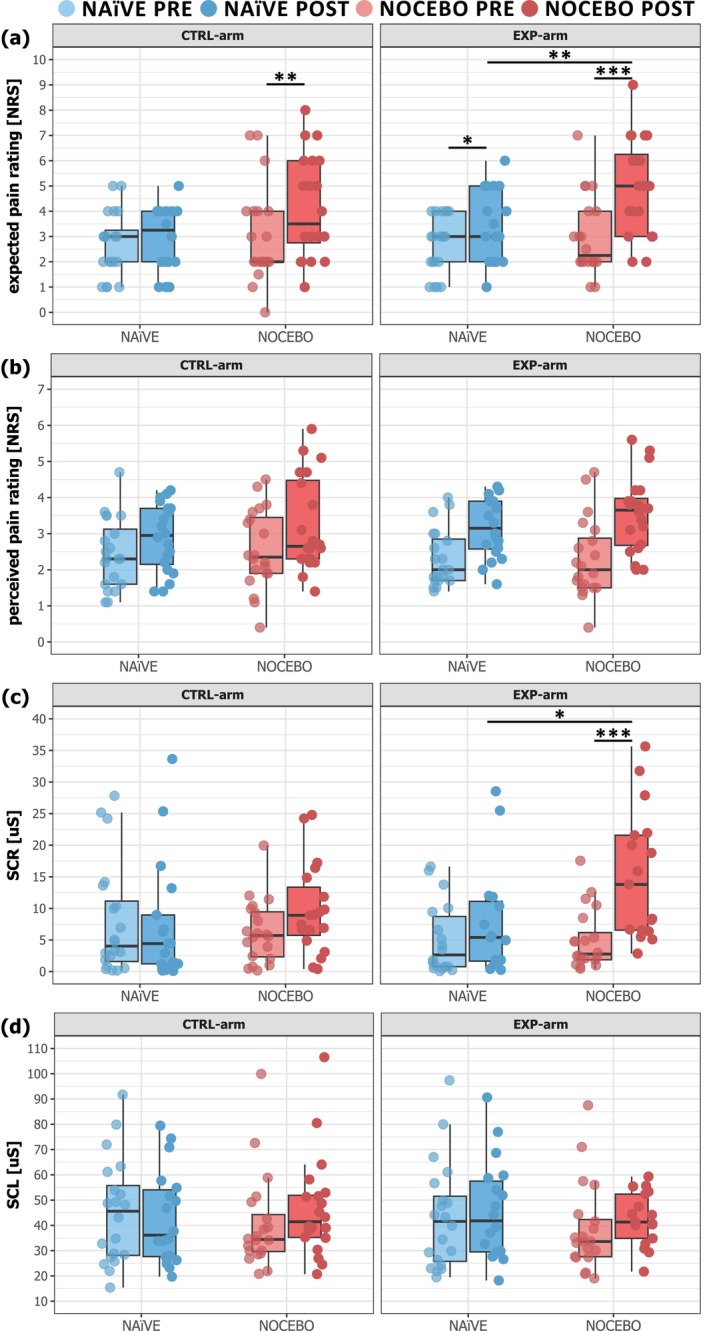
Boxplots of (a) expected pain rating, (b) perceived pain ratings, (c) phasic skin conductance responses (SCR) and (d) tonic skin conductance level (SCL) are shown for each group (NAïVE [blue] and NOCEBO [red]) and area (CTRL‐arm and EXP‐arm) for the PRE‐assessment (light) and the POST‐assessment (dark). *p < 0.05; **p < 0.01; ***p < 0.001.

In the EXP‐arm, the expected pain intensity increased for both the NOCEBO (PRE: 2.98 ± 1.56; POST: 4.90 ± 1.94; *N* = 20; *t* = 7.64; *p* < 0.001; Hedges' *g* = 1.0) and the NAïVE group (PRE: 2.70 ± 1.13; POST: 3.38 ± 1.42; *N* = 20; *t* = 2.68; *p* = 0.04; Hedges' *g* = 0.49) (Figure 3a). Moreover, the expected pain intensity in the POST assessment was higher in the NOCEBO compared to the NAïVE group (*N* = 40; *t* = 3.13; *p* = 0.01; Hedges' *g* = 0.88). In the CTRL‐arm, the expected pain intensity increased only for the NOCEBO (PRE: 3.03 ± 1.90; POST: 4.15 ± 2.03; *N* = 20; *t* = 4.05; *p* = 0.001; Hedges' *g* = 0.55) but not the NAïVE group (PRE: 2.70 ± 1.26; POST: 2.88 ± 1.32; *N* = 20; *t* = 0.63; *p* = 1.0; Hedges' *g* = 0.13).

#### 3.3.2 Perceived Pain Ratings Were Increased After the Heat Pain Model

For both the EXP‐arm and the CTRL‐arm only the main effect of ‘time’ was found to be significant (EXP: *F* = 53.07; *p* < 0.001; CTRL: *F* = 23.24; *p* < 0.001). Therefore, independent of the group (NOCEBO and NAïVE), the perceived pain ratings for pinprick stimuli were higher in the POST assessment for stimulation of the EXP‐arm and the CTRL‐arm.

#### 3.3.3 Combination of Sensitisation and Nocebo Instructions Increased Phasic Skin Conductance Response

**FIGURE 4:**
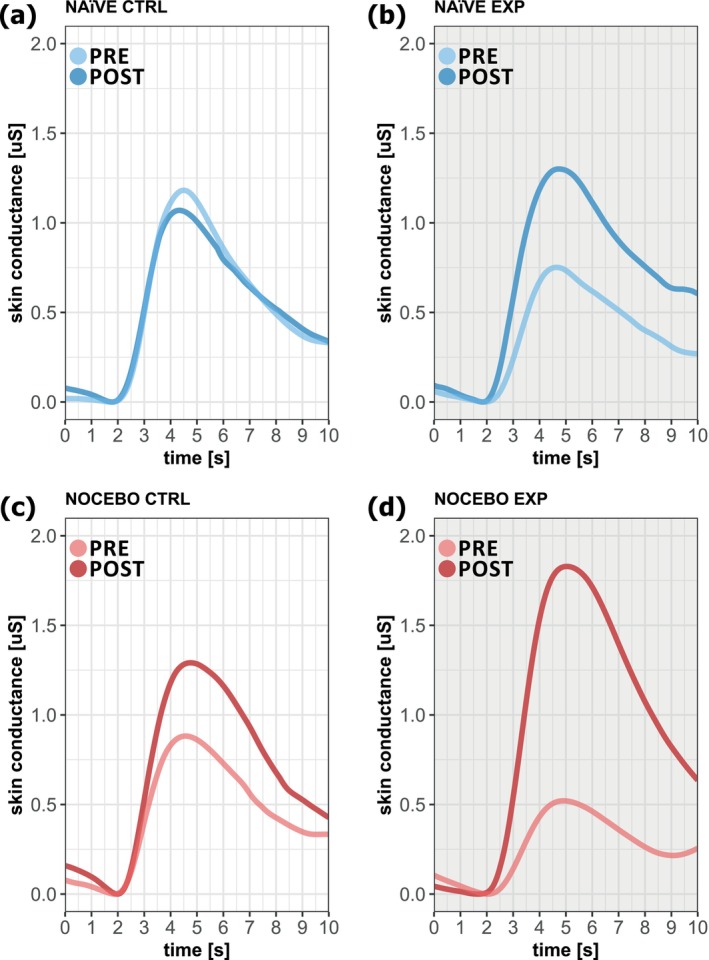
Average skin conductance recording of all participants within a 10‐s‐window after pinprick stimulation for each group. (a) CTRL‐arm of the NAïVE group (b) EXP‐arm of the NAïVE group (c) CTRL‐arm of the NOCEBO group (d) EXP‐arm of the NOCEBO group.

Figure 4 shows the average SCR of all participants after pinprick stimulation for each group and area (CTRL‐arm and EXP‐arm) for the PRE‐assessment and the POST‐assessment.

In contrast to the perceived pain, the nocebo instructions significantly affected the phasic SCR (‘time × group’; *F* = 5.89; *p* = 0.02) only in the EXP‐arm. Here the phasic SCR increased from PRE to POST assessment (PRE: 4.99 ± 4.72 μS; POST: 15.10 ± 10.15 μS; *N* = 17; *t* = 5.16; *p* < 0.001; Hedges' *g* = 1.19) while the phasic SCR of the NAïVE group did not change (PRE: 5.13 ± 5.66 μS; POST: 7.89 ± 8.75 μS; *N* = 15; *t* = 1.53; *p* = 0.54; Hedges' *g* = 0.52) (Figure 3c). Additionally, in the POST assessment, the phasic SCR was increased in the NOCEBO group compared to the NAïVE group (*N* = 32; *t* = 2.63; *p* = 0.04; Hedges' *g* = 0.74).

#### 3.3.4 Higher Tonic Skin Conductance Level After the Heat Pain Model

In both the EXP‐arm and the CTRL‐arm, only a significant main effect of ‘time’ was observed for tonic SCL during pinprick stimulation (EXP: *F* = 38.80; *p* < 0.001; CTRL: *F* = 12.50; *p* < 0.001). This reveals a general increased tonic background SCL in both groups (NOCEBO and NAïVE) after the experimental heat pain model, independent of the stimulated area (EXP‐arm and CTRL‐arm).

### 3.4 Increased Skin Conductance Level During Acoustic Stimulation

Neither for the phasic SCR nor the for tonic SCL a significant influence of the nocebo instructions on the change from PRE to POST was found (both ‘time × group’; *p* > 0.18). However, for the tonic background SCL a significant main effect ‘time’ was found (*F* = 14.36; *p* < 0.001). Similar to the noxious pinprick stimulation, this reveals a general and group‐independent increase in tonic SCL during the POST assessment.

### 3.5 Baseline Autonomic Measures Revealed Increased Sympathetic Outflow During the Heat Pain Model

**FIGURE 5:**
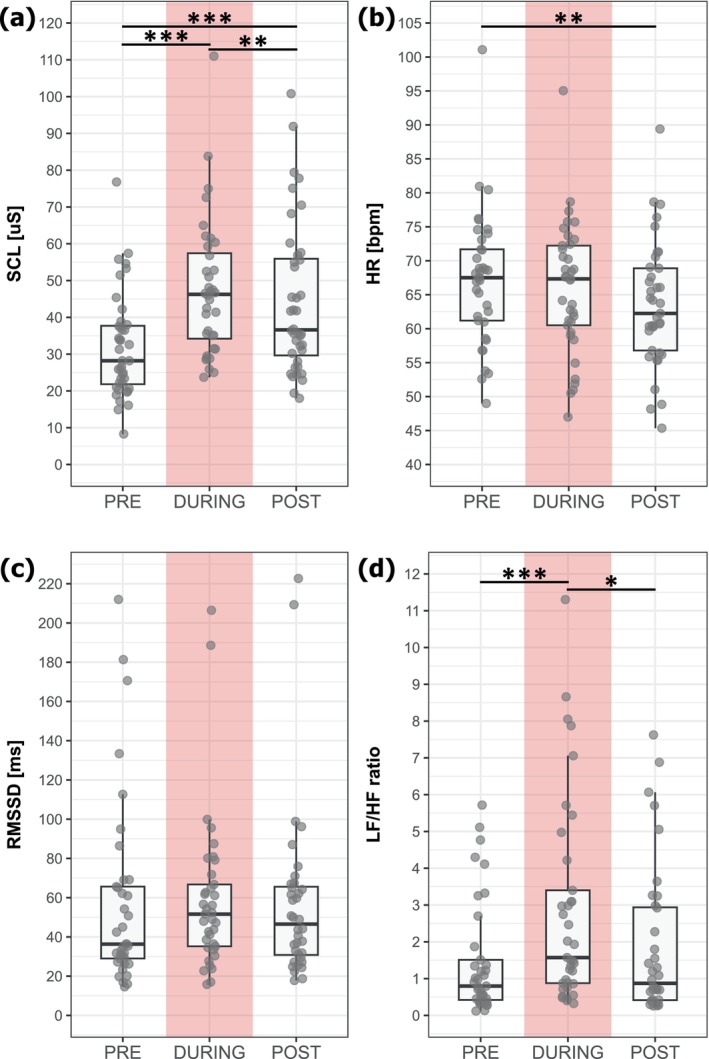
Boxplots of continuous autonomic measures, that is, (a) skin conductance level (SCL), (b) heart rate (HR), (c) time domain analysis (root mean square of successive differences [RMSSD]) and (d) frequency analysis of HR variability (ratio of low‐frequency power to high‐frequency power [LF/HF ratio]) before (PRE), during and after (POST) application of the heat pain model. *p < 0.05; **p < 0.01; ***p < 0.001.

The baseline tonic SCL increased from PRE (32.0 ± 14.15 μS) to DURING the heat pain model (47.52 ± 18.54 μS; *N* = 36; *t* = 12.17; *p* < 0.001; Hedges' *g* = 0.81) and decreased again from DURING to POST (44.46 ± 20.52 μS; *t* = −3.34; *N* = 36; *p* = 0.004; Hedges' *g* = −0.29) (Figure 5a). Baseline SCL was also increased in POST compared to PRE assessments (*N* = 40; *t* = 9.19; *p* < 0.001; Hedges' *g* = 0.57).

Three participants showed signs of possible heart arrhythmia (i.e., supraventricular extrasystoles) and were therefore excluded from the analysis of HR and HRV.

The overall HR decreased over the time course of the study visit (PRE: 67.18 ± 9.66 bpm vs. POST: 63.50 ± 9.20 bpm; *N* = 37; *t* = −3.28; *p* = 0.005; Hedges' *g* = −0.38) (Figure 5b). However, the HR did not change from PRE to DURING (65.92 ± 9.57 bpm; *N* = 37; *t* = −1.12; *p* = 0.80; Hedges' *g* = −0.13) or from DURING to POST (*N* = 37; *t* = −2.16; *p* = 0.10; Hedges' *g* = −0.25).

There were no differences in HRV measured as RMSSD between PRE (57.45 ± 47.97 ms), DURING (59.22 ± 39.84 ms) and POST (62.40 ± 53.12 ms; *N* = 37; all *p* = 1.0) (Figure 5c).

Lastly, similar to the SCL, the LF/HF ratio recorded at baseline increased from PRE (1.27 ± 1.51) to DURING the heat pain model (2.84 ± 3.35; *N* = 37; *t* = 3.87; *p* < 0.001; Hedges' *g* = 0.58) and decreased again from DURING to POST (1.66 ± 1.91; *N* = 37; *t* = −2.68; *p* = 0.03; Hedges' *g* = −0.38) (Figure 5d). The baseline LF/HF ratio did not differ between the PRE and POST assessments (*N* = 37; *t* = 1.16; *p* = 0.76; Hedges' *g* = 0.23).

## 4 Discussion

The aim of this study was to investigate the influence of verbally induced negative expectations on pain‐related autonomic responses after experimentally‐induced SMH. Our findings suggest that negative expectations alone do not suffice to modulate pain‐related phasic SCRs, but only when combined with experimentally‐induced SMH. Tonic background SCL, however, was generally increased after the applied heat pain model, independent of induced negative expectations or stimulus modality (i.e., non‐noxious acoustic stimulation). In the following paragraphs, we will discuss how both nociceptive sensitisation and negative expectations can influence pain‐related autonomic responses as well as the potential clinical relevance of our findings.

### 4.1 Negative Expectations Elevate Pain‐Related Autonomic Responses in the Area of SMH

The nocebo instructions led to increased expected pain ratings towards pinprick stimulation of the CTRL‐ and EXP‐arm. Previously, it has been shown that increased expected pain leads to enhanced SCR to noxious stimulation (Barnes et al. 2021; Koban and Wager 2016). However, in the present study, neither negative expectation (i.e., stimulation of the CTRL‐arm in the NOCEBO group) nor experimentally‐induced SMH alone (i.e., stimulation of the EXP‐arm in the NAïVE group) increased phasic SCRs. This is in contrast to previous studies which reported increased pain‐related autonomic responses after the induction of SMH (Salameh et al. 2022; Scheuren et al. 2020; van den Broeke et al. 2019). Opposed to these previous studies which adopted a fixed stimulation intensity (e.g., 256 mN pinprick), we matched the stimulus intensity to a perceived intensity of NRS 4/10 to account for stimulus‐associated arousal. Our findings are, therefore, in line with our recent study (Scheuren et al. 2023) in which stimulus intensity was also matched in perceived pain intensities. This highlights the effect of stimulus‐associated arousal, which could explain previous findings of enhanced phasic SCRs after experimentally‐induced SMH.

Only the combination of negative expectations and experimentally‐induced SMH (i.e., stimulation of the EXP‐arm in the NOCEBO group) led to an increase of phasic SCRs. While some studies provided evidence that SCRs to noxious stimuli are more closely related to applied stimulus intensity than perceived pain intensity (Loggia et al. 2011; Nickel et al. 2017), others showed that individual pain perception mediates pain‐related autonomic responses (Mischkowski et al. 2019; Tiemann et al. 2018, 2019). The latter argument partially explains our finding of increased SCRs in the NOCEBO group due to generally heightened pain ratings in the POST assessment.

However, the nocebo instructions alone did not lead to such an increase in phasic SCR, a finding that warrants further explanation. While the expected pain rating for the CTRL‐arm increased after the nocebo instructions, those for the EXP‐arm showed a larger increase. Previously it was demonstrated that SCRs only increased when participants expected more pain (Koban and Wager 2016). The higher increase of expectancy ratings on the EXP‐arm might have been needed to translate into a significant change in SCR. This would also reflect findings of Reicherts et al. 2016 where changes in SCR were only found when expectancy of participants was manipulated with verbal instructions combined with a conditioning trial (i.e., learned negative expectation). Further, evidence suggests that nocebo effects (i.e., expectancy manipulation on pain ratings) become more robust by reinforcing them over multiple conditioning trials (Colloca et al. 2010). Therefore, it is possible that the painful experience of the heat pain model on the EXP‐arm of the NOCEBO group strengthened the negative expectations towards the pinprick stimulation. This hypothesised effect of the heat pain model on the expected pain rating might also explain the slight increase of expected pain in the EXP‐arm of the NAïVE group. Additionally, similar to induced SMH, nocebo manipulations do lead to an amplification of spinal pain signals (Geuter and Büchel 2013). The interplay between bottom‐up (SMH) and top‐down (nocebo) manipulation consequently led to a significant increase in phasic SCR driven by mutual central facilitatory processes. On these grounds, increased SCR found in chronic pain cohorts might be a combination of sensitisation‐related pain hypersensitivities (Latremoliere and Woolf 2009) and hypervigilance (Rogers and Farris 2022).

### 4.2 Autonomic Measures Reflect Sympathetic Arousal During the Heat Pain Model

Tonic background SCL increased during the application of the heat pain model and remained enhanced up to 20 min after the induction of SMH. Additionally, the LF/HF ratio was increased during the heat pain model but decreased thereafter to similar values measured in the PRE assessment. Both the tonic SCL (Hu et al. 2018) and the increase of the LF/HF ratio (Shaffer and Ginsberg 2017) reflect sympathetic activity. Enhanced sympathetic activity during the pain experience, measured as SC (Storm 2008) and HRV (Forte et al. 2022; Koenig et al. 2014) is well established. Therefore, the increase in SCL and LF/HF ratio depicts a high sympathetic arousal during the heat pain model. Interestingly, only SCL stayed elevated after the heat pain model (i.e., higher SCL in the baseline measurement POST compared to PRE). Similar findings have been shown previously, in which recovery of sympathetic activity (measured as SCL) was slower compared to recovery of parasympathetic activity (measured as respiratory sinus arrhythmia) after a social stress test (Ho et al. 2020). As the LF/HF ratio is influenced by both the sympathetic and parasympathetic systems, the fast recovery of the LF/HF ratio can be explained by the sharp parasympathetic rebound after a stressful event (Mezzacappa et al. 2001), while the purely sympathetic measure of SCL indicates long‐lasting sympathetic arousal after the heat pain model.

On the contrary, we observed no change in RMSSD, which reflects parasympathetic activity (Thomas et al. 2019), during exposure to the heat pain model. Further, the HR did not increase during the heat pain model and even decreased thereafter. This decrease could potentially be explained by the 20 min resting phase after the heat pain model, reflecting similar mechanisms as in the cardio‐deceleration (i.e., parasympathetic reactivation and sympathetic withdrawal) after exercise (Michael et al. 2017).

### 4.3 Heat Pain Model Induces General Autonomic Arousal

Generally increased background SCL was found in the POST assessment, independent of the group (NOCEBO and NAïVE), stimulated area (EXP‐arm and CTRL‐arm) and stimulation modality (pinprick and acoustic). This indicates heightened sympathetic arousal as depicted during induction of SMH. These findings are in line with our previous study (Scheuren et al. 2023) in which increased tonic SCL was found after noxious heat and pinprick stimulation of the sensitised area. Novel, however, is the observed heightened tonic background SCL in the absence of a noxious stimulation of the area of SMH (i.e., CTRL‐arm or acoustic stimulation). This indicates that the high sympathetic activation during the heat pain model might lead to a general increase in autonomic responsiveness, previously described as a state of autonomic priming (Scheuren et al. 2023). Thus, within this state of autonomic priming, the autonomic system might be more susceptible to a multitude of stimuli, including but not restricted to noxious input. Interestingly, increased autonomic arousal has been postulated as a mechanism underlying the persistence of nocebo hyperalgesia (Colagiuri and Quinn 2018). Therefore, autonomic priming, induced through the heat pain model, might also have facilitated nocebo responses. Altogether, in our experiment, we predominantly observed general induced arousal through the experimental heat pain model, while the nocebo effect acts more specifically on noxious stimuli.

### 4.4 Limitations

This study provides first insights into the interplay between negative expectations and experimentally‐induced SMH on pain‐related autonomic readouts. There are some limitations worth noting: Firstly, several recordings of phasic SCR and tonic SCL had to be excluded from our analysis due to the SC exceeding the recording window of −40 μS to +40 μS. In some cases, this led to smaller sample sizes for within group (anticipated *N* = 20) and between group (anticipated *N* = 40) comparisons than planned. Secondly, due to the verbal nocebo instructions, the experimenters were not blinded which participants were assigned to the NAïVE and NOCEBO group. Thirdly, the participants were only allocated to either the NAïVE or NOCEBO group. Therefore, we cannot generalise the effects of the nocebo instructions for the whole study population. Lastly, no control condition, for example, non‐painful model, without inducing SMH was investigated. Thus, we cannot fully exclude effects of the heat pain model outside of the area of SMH, such as on the CTRL‐arm.

## 5 Conclusion

The findings provided in this study indicate that both induced SMH and negative expectations influence pain‐related autonomic responses, and they do so in an additive manner, specifically on pinprick‐evoked phasic SCR. New considerations for the use of pain‐related autonomic readouts to detect pain hypersensitivities in individuals with chronic pain may be inferred from this study. Without fully accounting for negative expectation towards stimuli perceived as painful, pain‐related autonomic readouts likely represent a combination of both bottom‐up (i.e., sensitization) and top‐down (expectation) mechanisms. Pain catastrophising, hypervigilance and anxiety are strongly linked to different chronic pain conditions (Rogers and Farris 2022) which could lead to the misinterpretation and/or exaggeration of a potentially threatening situation, such as a noxious stimulation. Therefore, it is of paramount importance to account for the effects of negative expectations towards noxious stimuli when assessing individuals with chronic pain. Reducing anticipatory anxiety of participants and patients as much as possible, especially during the assessment of pain‐related autonomic responses, is thus crucial. This includes, but is not limited to, providing a calm and safe environment, as well as reducing possible anxiety with clear instructions and familiarisation of the procedure. Further, blinding participants towards an applied noxious stimulus and randomising the time interval between stimuli would lower the predictability of incoming stimulation, thereby lowering the enhancement of pain‐related autonomic responses through expectation. Nevertheless, pain is, by nature, a multidimensional experience. While it is necessary to control for external factors as much as possible, the effects of negative expectation likely cannot be fully accounted for when investigating pain‐related autonomic responses in the context of pain hypersensitivities. However, being aware of negative expectation as a contributing factor in altered pain‐related autonomic measures is important and may lead to improved understanding of maladaptive changes in pain processing in chronic pain cohorts.

## Author Contributions

F.A. contributed substantially to the data acquisition, analysis, interpretation and drafted the manuscript. J.R. contributed to the study conception and design, interpretation of results and revised the manuscript. T.E. contributed data acquisition and revised the manuscript. P.S.S. substantially contributed to the study conception and design, data analysis, interpretation of results and revised the manuscript. M.H. made substantial contributions to the study conception and design, data analysis, interpretation of results and revised the manuscript. All authors approved the final version of the manuscript.

## Conflicts of Interest

The authors declare no conflicts of interest.

## Supporting information


Appendix S1.
