# Supplementary material for: Nocebo Effect on Pain‐Related Autonomic Responses in a State of Experimentally‐Induced Sensitization
Source: Eur J Pain. 2025 Apr 18;29(5):e70029. doi: 10.1002/ejp.70029 (PMC12008480; doi:10.1002/ejp.70029)
Supplement: Supplementary file 1 — Appendix S1. [file EJP-29-0-s001.pdf]

CTRL-arm

tableS1 A)	time		group		time * group	
	F-value	p-value	F-value	p-value	F-value	p-value
a) pain expected	10.96	0.002	2.69	0.11	5.85	0.02
b) pain perceived	23.24	<0.001	0.53	0.47	-	-
c) phasic SCR	7.88	0.008	0.04	0.84	-	-
d) tonic SCL	12.5	0.001	0.19	0.66	-	-

tableS1 B)			POST vs. PRE		
			t-value	p-value	Hedges' g
a) pain expected	NAïVE	N=20	0.63	1	0.13
	NOCEBO	N=20	4.05	0.001	0.55

tableS1 C)			NOCEBO vs. NAïVE		
			t-value	p-value	Hedges' g
a) pain expected	PRE	N=40	0.62	1	0.2
	POST	N=40	2.43	0.08	0.73

Supplementary Table S1:

tS1 A) Model statistics for comparison of expected pain ratings, percieved pain rating, phasic skin conductance responses (SCR) and tonic skin conductance level (SCL) during pinprick stimulation of the CTRL-arm.

tS1 B) Post-hoc comparisons of expected pain ratings during pinprick stimulation between timepoints (POST vs. PRE).

tS1 C) Post-hoc comparisons of expected pain ratings during pinprick stimulation between groups (NOCEBO vs. NAïVE).

EXP-arm

tableS2 A)	time		group		time * group	
	F-value	p-value	F-value	p-value	F-value	p-value
a) pain expected	53.21	<0.001	3.93	0.05	12.3	0.001
b) pain perceived	53.07	<0.001	0.4	0.53	-	-
c) phasic SCR	21.79	<0.001	2.7	0.11	5.89	0.02
d) tonic SCL	38.8	<0.001	0.34	0.56	-	-

tableS2 B)			POST vs. PRE		
			t-value	p-value	Hedges' g
a) pain expected	NAïVE	N=20	2.68	0.04	0.49
	NOCEBO	N=20	7.64	<0.001	1
a) phasic SCR	NAïVE	N=15	1.53	0.54	0.52
	NOCEBO	N=17	5.16	<0.001	1.19

tableS2 C)			NOCEBO vs. NAïVE		
			t-value	p-value	Hedges' g
a) pain expected	PRE	N=40	0.56	1	0.2
	POST	N=40	3.13	0.01	0.88
a) phasic SCR	PRE	N=38	-0.06	1	-0.03
	POST	N=32	2.63	0.04	0.74

Supplementary Table S2:

tS2 A) Model statistics for comparison of expected pain ratings, percieved pain rating, phasic skin conductance responses (SCR) and tonic skin conductance level (SCL) during pinprick stimulation of the EXP-arm.

tS2 B) Post-hoc comparisons of expected pain ratings and phasic skin conductance responses (SCR) during pinprick stimulation between timepoints (POST vs. PRE).

tS2 C) Post-hoc comparisons of expected pain ratings and phasic skin conductance responses (SCR) during pinprick stimulation between groups (NOCEBO vs. NAïVE).

Acoustic Stimulation

tableS3)	time		group		time * group	
	F-value	p-value	F-value	p-value	F-value	p-value
a) phasic SCR	2.04	0.16	0.03	0.86	-	-
b) tonic SCL	14.36	<0.001	0.32	0.58	-	-

Supplementary Table S3:

tS3) Model statistics for comparison of phasic skin conductance responses (SCR) and tonic skin conductance level (SCL) during acoustic stimulation.

Mechanical Hyperalgesia

tableS4 A)	time		area		time * area	
	F-value	p-value	F-value	p-value	F-value	p-value
a) MPT	21.29	<0.001	10.32	0.002	4.24	0.04
b) Intensity	82.79	<0.001	82.79	<0.001	61.45	<0.001

tableS4 B)			POST vs. PRE		
			t-value	p-value	Hedges' g
a) MPT	CTRL-arm	N=40	-1.81	0.29	-0.26
	EXP-arm	N=40	-4.72	<0.001	-1.1
b) Intensity	CTRL-arm	N=40	-0.89	1	-0.17
	EXP-arm	N=40	-11.98	<0.001	-1.67

tableS4 C)			EXP-arm vs.CTRL-arm		
			t-value	p-value	Hedges' g
a) MPT	PRE	N=40	-0.82	1	-0.12
	POST	N=40	-3.73	0.001	-1.29
b) Intensity	PRE	N=40	-0.89	1	-0.17
	POST	N=40	-11.98	<0.001	-1.76

Supplementary Table S4:

tS4 A) Model statistics for comparison of mechanical pain threshold (MPT) and stimulation intensity needed to reach NRS 4/10.

tS4 B) Post-hoc comparisons of mechanical pain threshold (MPT) and stimulation intensity needed to reach NRS 4/10 between timepoints (POST vs. PRE).

tS4 C) Post-hoc comparisons of mechanical pain threshold (MPT) and stimulation intensity needed to reach NRS 4/10 between stimulated areas (EXP-arm vs. CTRL-arm).

Baseline Comparison

tableS5 A)	time	
	F-value	p-value
a) SCL	81.37	<0.001
b) HR	5.57	0.006
c) RMSSD	0.39	0.68
d) LF/HF ratio	7.86	<0.001

tableS5 B)		DURING vs.PRE		
		t-value	p-value	Hedges' g
a) SCL	N=36	12.17	<0.001	0.81
b) HR	N=37	-1.12	0.8	-0.13
c) LF/HF ratio	N=37	3.87	<0.001	0.58

tableS5 C)		DURING vs.POST		
		t-value	p-value	Hedges' g
a) SCL	N=36	3.34	0.004	0.29
b) HR	N=37	2.16	0.1	0.25
c) LF/HF ratio	N=37	2.68	0.03	0.38

tableS5 D)		POST vs.PRE		
		t-value	p-value	Hedges' g
a) SCL	N=40	9.19	<0.001	0.57
b) HR	N=37	-3.28	0.005	-0.38
c) LF/HF ratio	N=37	1.16	0.76	0.23

Supplementary Table S5:

tS5 A) Model statistics for comparison of tonic skin conductance level (SCL), heart rate (HR) and HR variability measurements (root mean square of successive differences (RMSSD) and the ratio of low-frequency power to high-frequency power (LF/HF ratio).

tS5 B-D) Post-hoc comparisons of SCL, HR and LF/HF ratio between timepoints (PRE, DURING and POST).

Exclusion of Autonomic Measures

tableS6 A)		SCR		SCL	
		PRE	POST	PRE	POST
a) Pinprick	NAïVE CTRL-arm	N=0	N=3	N=0	N=2
	NAïVE EXP-arm	N=2	N=5	N=0	N=2
	NOCEBO CTRL-arm	N=0	N=1	N=0	N=0
	NOCEBO EXP-arm	N=0	N=3	N=0	N=3
a) Acoustic	NAïVE	N=0	N=2	N=0	N=2
	NOCEBO	N=0	N=1	N=0	N=0

tableS6 B)	PRE	DURING	POST
a) SCL	N=0	N=4	N=0
b) HR measures	N=3	N=3	N=3

Supplementary Table S6:

tS6 A) Exclusion of phasic skin conductance response (SCR) and tonic skin conductance level (SCL) recodings due to exceeding the recording window of ±40uS during pinprick and acoustic stimulation.

tS6 B) Exclusion of tonic SCL recodings due to exceeding the recording window of ±40uS and heart rate (HR) measures due to signs of possible heart arrythmia during baseline measurements.
